# Intravascular Ultrasound for Intracranial and Extracranial Carotid Artery Stent Placement

**DOI:** 10.7759/cureus.732

**Published:** 2016-08-10

**Authors:** Ahmad S Hussain, Namath S Hussain

**Affiliations:** 1 Vascular Surgery, Detroit Medical Center; 2 Department of Neurosurgery, Loma Linda University Medical Center

**Keywords:** intravascular ultrasound, internal carotid artery dissection

## Abstract

Intravascular ultrasound (IVUS) can provide valuable information regarding endoluminal morphology. We present the first description of IVUS-guided intracranial and extracranial carotid artery stent placement for arterial dissection. A 41-year-old female with a sudden-onset headache and blurred vision underwent a computed tomography (CT) angiogram imaging that revealed bilateral carotid artery dissections (BCAD) and a left vertebral artery dissection (VAD). Endovascular treatment (EVT) of a long segment right carotid artery dissection (CAD) was performed employing two Carotid WALLSTENT™ Monorails™ (8 x 36 mm, 10 x 31 mm) (Boston Scientific, Marlborough, MA). With the help of the IVUS, the distal stent was placed up to the petrous carotid artery, followed by the placement of the second stent in the immediate proximal location with some overlap that extended down to the carotid artery bulb. Intraoperative angiography and post-stenting IVUS revealed excellent stent placement with good resolution of the dissection and good luminal patency with pseudolumen obliteration. Stent use for intracranial circulation dissections will continue to be a favorable option given the decreased morbidity of endovascular therapy in this location. As endovascular surgeons become more facile with the use of IVUS, using it as a guide for stent placement and post-stenting confirmation will help them to ensure proper positioning and improved patency rates.

## Introduction

Conventional digital subtraction angiography (DSA), the current gold standard for intracranial vascular imaging, is hampered by its inability to clearly demonstrate intraluminal plaque composition and vessel morphology. Such an understanding is important because of the attendant risks of rupture and malpositioning of stents and coils in the thin-walled, tortuous intracranial circulation.

IVUS can provide valuable information regarding endoluminal morphology. IVUS was first developed as an adjunct for use during percutaneous endovascular coronary artery interventions. Its ease of use and unique capability to provide real-time endoluminal views that conventional angiography cannot provide have made it a useful tool in difficult cases where intraluminal vessel morphology can play an important role in deciding which therapy to undertake. It is also useful in assisting with the intervention and post-intervention confirmation. IVUS had not been previously used to assist in combined intracranial and extracranial carotid artery stenting.

The results of the Guidance by Ultrasound Imaging for Decision Endpoints (GUIDE) trial published by the American College of Cardiology (ACC) have provided good longitudinal data regarding intervention results and the factors that predispose to restenosis, such as postangioplasty lumen diameter, area, and plaque percentage [1]. Recent advancements in vascular stents have made it possible to deploy stents with the aim of treating intracranial atherosclerotic disease (IAD). However, the risk of arterial rupture is high because of small luminal diameter and the lack of surrounding supportive adventitia. The risks of stenting are also higher when the vessel is long and tortuous. These risks can be compounded when the procedure is aimed at treating not only stenosis due to atherosclerosis deposits but also large-based dissection flaps that occlude the lumen.

IVUS provides the information necessary to assess the intraluminal in vivo morphology to determine when a lesion is safe to undergo stent placement and real-time imaging (RTI) to ensure proper treatment of the dissection flap [2]. We present the first description of IVUS-guided intracranial and extracranial carotid artery stent placement for arterial dissection. IVUS was used in the placement of two Carotid WALLSTENT monorails (8 x 36 mm, 10 x 31 mm). This technical note illustrates how IVUS can be used to identify multiple areas of dissection and also determine the size and length of the lesions. This information will help the practitioner make a more informed decision as to whether one or more stents are required to treat the abnormality.

## Case presentation

The patient was seen in inpatient consultation as requested by the vascular surgery service. The patient provided written consent for the endovascular and imaging procedures. Arterial access was obtained via a femoral approach. A 5-French angled Cordis guide catheter (Cordis Corporation, Fremont, CA) with a 0.035 Terumo guidewire (Terumo Medical Corporation, Somerset, NJ) were used. Systemic anticoagulation was undertaken through heparin to maintain an activated coagulation time between 200 and 300 seconds. The patient also received preoperative aspirin and Plavix. A 3.4 Fr 20 MHz IVUS probe (Volcano Corporation, San Diego, CA) was used for data collection. All images were displayed and recorded on an IVUS portable workstation (Volcano Corporation, San Diego, CA).

**Figure 1:**
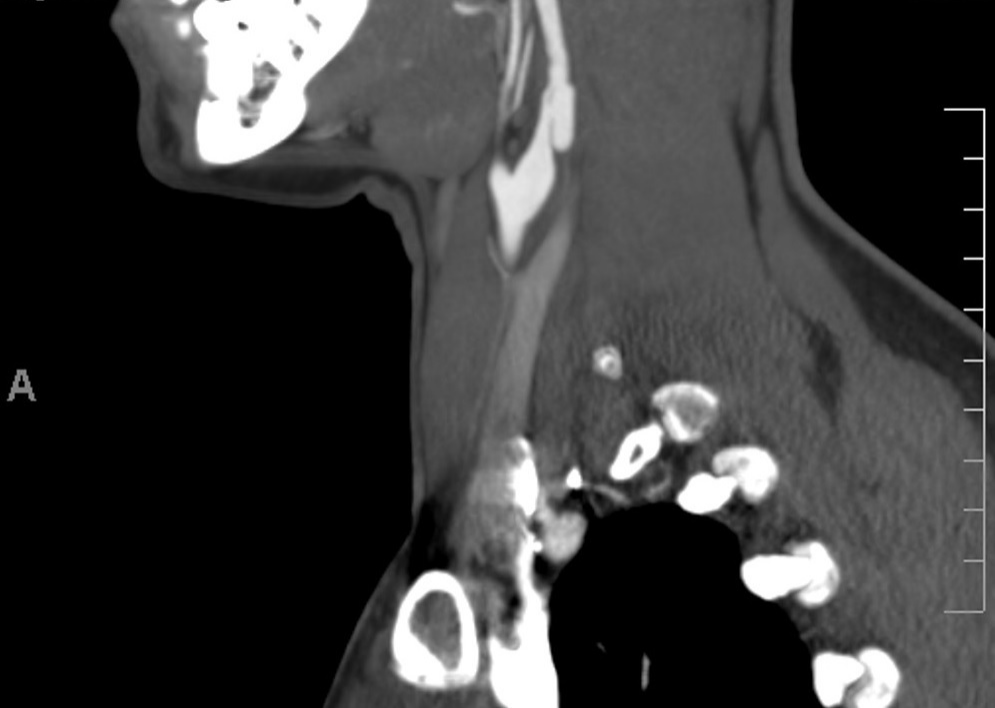
A CT angiogram showing carotid dissection

While exercising on an elliptical machine, a 41-year-old female experienced a popping sound in her left ear followed by a headache at the left occipital aspect of her skull base and blurry vision. She presented to the emergency department where a CT angiogram revealed bilateral carotid dissections as shown in Figure 1 and a left vertebral artery dissection.

A CT of the abdomen and pelvis also revealed dissection of the left renal artery. She was observed and started on Coumadin and subsequently discharged. She returned to the hospital with continued symptoms and was subsequently transferred to our service for definitive care. Her past medical history was significant for lupus and fibromyalgia. She had no history of Ehlers-Danlos syndrome, Marfan syndrome, vasculitis, or fibromuscular dysplasia.

The patient was started on aspirin, Plavix, and a heparin drip preoperatively. The patient's platelet function assays revealed a 46% platelet inhibition of function from Plavix and an aspirin platelet function test value of 500, which was therapeutic. The patient’s platelet count was 183, creatinine was 1.0, and partial thromboplastin time (PTT) was 68.

**Figure 2:**
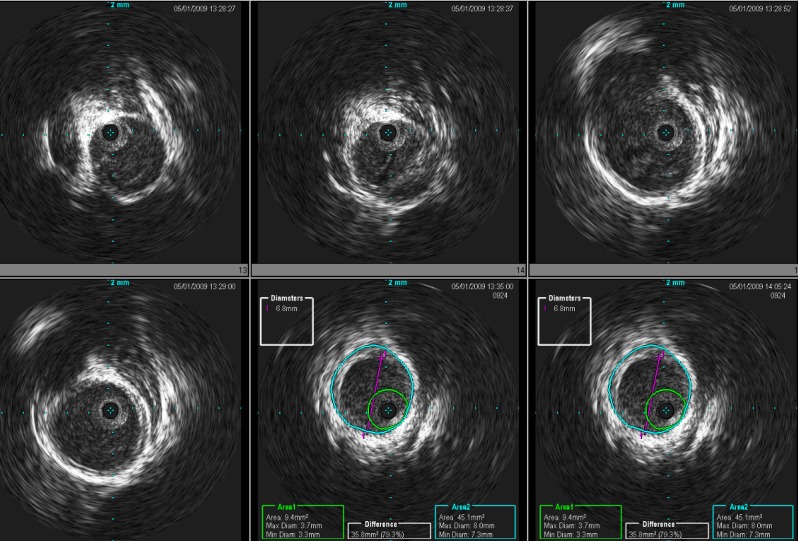
A pre-stent IVUS showing dissection flap and compromised luminal diameter

Formal four-vessel cerebral angiography confirmed the findings on her previous CT angiogram. An angiography was undertaken with a 300-cm synchro 0.014-inch wire (Boston Scientific/Precision Vascular Systems, Natick, MA) and a Cordis sheath (Cordis Corporation, Fremont, CA). The microcatheter was removed and the IVUS catheter was advanced into the petrous carotid artery. IVUS imaging was performed as the catheter was withdrawn slowly over the wire to document the extent of the dissection by correlating the fluoroscopic/roadmap positioning of the probe with the IVUS image as shown in Figure 2.

Two Carotid WALLSTENT Monorails (8 x 36 mm, 10 x 31 mm) were placed in the internal carotid artery spanning from the petrous internal carotid artery with some overlap down to the carotid bulb. With the help of the IVUS, the distal stent was placed up to the petrous carotid artery, followed by the placement of the second stent in the immediately proximal location with some overlap extending down to the carotid bulb.

**Figure 3:**
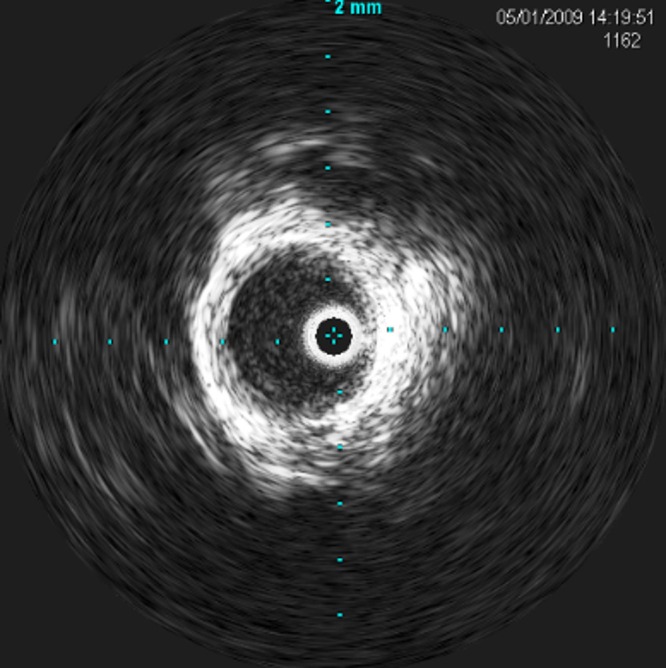
A post-stent IVUS showing restored endoluminal space

No balloon was used. Repeated angiography demonstrated full recanalization of the right internal carotid artery. Intraoperative angiography and post-stenting IVUS revealed excellent stent placement with good resolution of the dissection and good luminal patency with pseudolumen obliteration throughout all dissection segments as shown in Figure 3.

The patient was continued on aspirin (325 mg) and Plavix (75 mg) and was kept on a heparin drip at 600 u/hr overnight. She remained neurologically intact and was discharged home the following week.

## Discussion

Advances in coronary angiography have led to significant improvements in percutaneous interventions to an extent that intraluminal ultrasonography has become a standard tool in the cardiologist’s armamentarium. Conventional DSA angiography, the current gold standard for intracranial vascular imaging, is hampered by its inability to clearly demonstrate intraluminal plaque composition and vessel morphology. Such an understanding is important because of the attendant risks of rupture and malpositioning in the thin-walled, tortuous intracranial circulation.

IVUS allowed us to recognize that there were two separate dissections and not one as was observed on CT angiography. It also allowed us to accurately assess stent apposition and vascular patency with obliteration of the pseudolumen.

Intracranial IVUS helps to more accurately identify intraluminal pathology, identify risk factors for restenosis and assure full dissection coverage with the stent. It can also be used to define aneurysm neck and arteriovenous malformation (AVM) feeder vessel morphology and vessel composition to differentiate whether the vessel wall abnormality is calcification or thrombus [3-4].

The key impediment to IVUS imaging in the intracranial circulation has been a technical one [5]. However, with the improvement in the next generation probes and self-expanding stents, IVUS can assume the role of a useful adjunct during stent placement and delivery. Further advances will lead to better apposition of dissection flaps and a more clear delineation of inner vessel surface characteristics.

## Conclusions

Stent use for intracranial circulation dissections will continue to be a favorable option given the decreased morbidity of endovascular therapy in this location. The capability of IVUS to provide real-time endoluminal views, which conventional DSA cannot provide, has made it a useful tool in difficult cases where intraluminal vessel morphology can play an important role in deciding which therapy to undertake. It is also useful in assisting with the intervention as well as post-intervention confirmation. This might help to ensure proper positioning and improved patency rates. Intracranial IVUS helps to more accurately identify intraluminal pathology, identify risk factors for restenosis, and assure full dissection coverage with the stent. It can also be potentially used to better define aneurysm neck and AVM feeder vessel morphology and vessel composition to differentiate whether the vessel wall abnormality is calcification or thrombus.
